# Molecular, Morphological and Electrophysiological Differences between Alpha and Gamma Motoneurons with Special Reference to the Trigeminal Motor Nucleus of Rat

**DOI:** 10.3390/ijms25105266

**Published:** 2024-05-12

**Authors:** Youngnam Kang, Mitsuru Saito, Hiroki Toyoda

**Affiliations:** 1 Department of Behavioral Physiology, Graduate School of Human Sciences, Osaka University, Osaka 565-0871, Japan; 2 Department of Oral Physiology, Graduate School of Medical and Dental Sciences, Kagoshima University, Sakuragaoka 8-35-1, Kagoshima 890-8544, Japan; mtrsaito@dent.kagoshima-u.ac.jp; 3 Department of Oral Physiology, Graduate School of Dentistry, The University of Osaka, Osaka 565-0871, Japan

**Keywords:** α-motoneuron, γ-motoneuron, estrogen-related receptor 3, NeuN, LTS, early outward rectification, Ca^2+^-activated cationic channel-mediated ADP, flufenamic-acid-sensitive ADP

## Abstract

The muscle contraction during voluntary movement is controlled by activities of alpha- and gamma-motoneurons (αMNs and γMNs, respectively). In spite of the recent advances in research on molecular markers that can distinguish between αMNs and γMNs, electrophysiological membrane properties and firing patterns of γMNs have remained unknown, while those of αMNs have been clarified in detail. Because of the larger size of αMNs compared to γMNs, blindly or even visually recorded MNs were mostly αMNs, as demonstrated with molecular markers recently. Subsequently, the research on αMNs has made great progress in classifying their subtypes based on the molecular markers and electrophysiological membrane properties, whereas only a few studies demonstrated the electrophysiological membrane properties of γMNs. In this review article, we provide an overview of the recent advances in research on the classification of αMNs and γMNs based on molecular markers and electrophysiological membrane properties, and discuss their functional implication and significance in motor control.

## 1. Introduction

The muscle contraction during voluntary movement is precisely regulated by activities of alpha- and gamma-motoneurons (αMNs and γMNs, respectively), and the activations of αMNs and γMNs occur almost simultaneously [1,2]. This phenomenon is known as α–γ coactivation, which is necessary to compensate for mechanical unloading caused by the shortening of extrafusal muscles so that the stretch receptors of the muscle spindle can remain sensitive [3]. The α–γ coactivation also plays a critical role in voluntary isometric contraction of jaw-closing muscles during the slow jaw-closing phase [4] as well as in that of human lumbrical muscles [5]. The spindle Ia impulse is known to be involved in producing 30–40% of the isometric contraction of leg and hand muscles [6,7]. In agreement with these reports, it was previously demonstrated that the muscle spindle Ia activity caused by γMN activity is involved in increasing the isometric contraction of masseter muscles [4], during which αMNs are orderly recruited [8]. Thus, γMNs play an important role in regulating the isometric contraction. However, compared to αMNs, very little information is available regarding the firing pattern or excitability of γMNs, and the intrinsic membrane properties of γMNs have not been well studied for a long time regardless of whether in in-vivo or in-vitro preparations. This has been partly due to the absence of molecular markers to differentially identify αMNs and γMNs.

Recently, molecular markers to identify αMNs and γMNs have been found. Estrogen-related receptor 3 (Err3) is one of the nuclear receptors that contributes to the generative differentiation of γMNs [9]. In a spinal motor nuclei of mice, it was shown that Err3 is highly expressed in γMNs but not in αMNs, whereas NeuN, the neuronal DNA-binding protein, is highly expressed in αMNs but not in γMNs [9]. Using the two molecular markers, electrophysiological membrane properties have been studied in spinal MNs [10] and trigeminal jaw-closing MNs [11], while such studies still remain very few in the literature. In this review, we discuss the molecular markers of αMNs and γMNs and the membrane properties/firing patterns of αMNs and γMNs identified using such markers, together with their distribution and morphologies in the spinal cord and the dorsal part of the trigeminal motor nucleus (TMN). Our comprehensive literature search was conducted on PubMed/MEDLINE databases, with a language restriction to English. The keywords used for the research were “molecular markers”, “αMNs” and “γMNs”, together with “membrane properties” and “firing pattern”.

## 2. Molecular Markers of αMNs and γMNs

**Figure 1:**
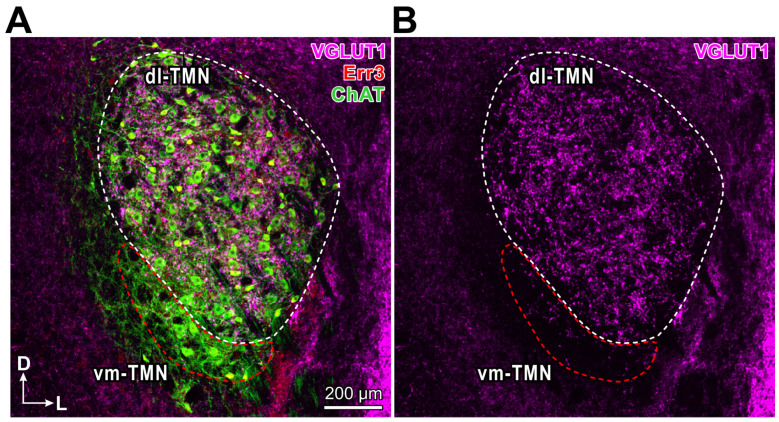
Immunohistochemical expression of VGLUT1, Err3 and choline acetyltransferase (ChAT) in the rat TMN. (**A**) A confocal image shows immunohistochemical expression of VGLUT1 (pink), Err3 (red) and ChAT (green). Fluorescence signals for VGLUT1/Err3/ChAT were obtained using Alexa Fluor 649, TSA Cyanine (Cy3) and Alexa Fluor 488, respectively. Dorsolateral- and ventromedial-TMN: dl-TMN and vm-TMN, respectively. (**B**) Differential distribution of VGLUT1-immunreactive terminals between dl-TMN (where jaw-closing MNs are located, white interrupted line) and vm-TMN (where jaw-opening MNs are located, red interrupted line). Scale bar in (**A**) applies to (**B**). Adapted from [12].

αMNs and γMNs are intermingled in motor nuclei in the spinal cord [12] as well as in the TMN ([13]: Figure 1). In a spinal motor nuclei of mice, the transcription factor Err3, an orphan nuclear hormone receptor, has been demonstrated to be highly expressed in γMNs but not αMNs, whereas the neuronal DNA-binding protein NeuN labels αMNs but not γMNs [9]. Immunohistochemical staining for choline acetyltransferase (ChAT) in combination with Err3 and/or NeuN in the spinal motor nuclei revealed the presence of two populations of small and large MNs. The small MNs were identified as a population of γMNs based on the Err3-positive and NeuN-negative expression, while the large MNs were identified as that of αMNs based on the NeuN-positive and Err3-negative expression [9]. Thus, NeuN and Err3 were expressed exclusively in αMNs and γMNs in the lumbar spinal cord, respectively.

**Figure 2:**
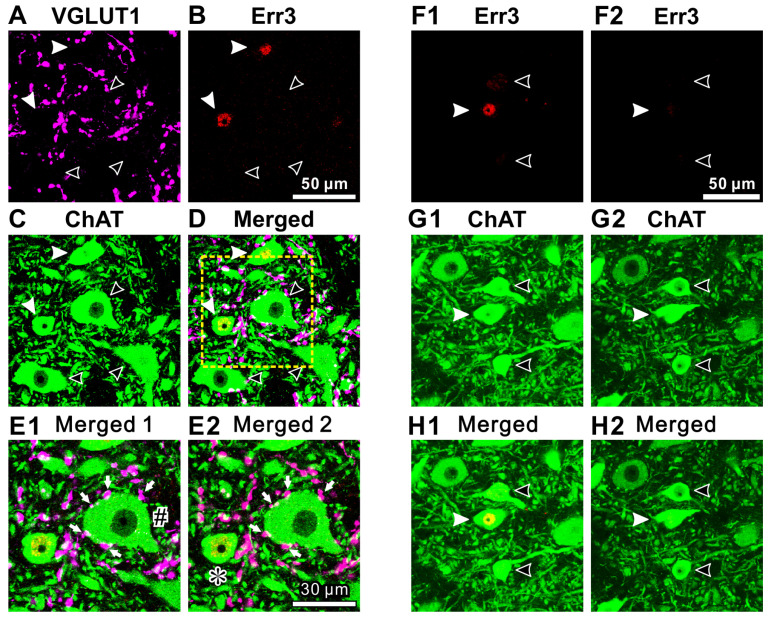
Immunohistochemical expression of VGLUT1, Err3 and ChAT in the dorsolateral TMN MNs. (**A**–**D**) Z-stack images of two successive confocal sections (1 μm apart) that were obtained by the triple immunofluorescence staining for VGLUT1 (pink), Err3 (red) and ChAT (green) ((**A**–**C**), respectively). (**D**) shows a merged fluorescence image of (**A**–**C**). Filled and open arrowheads indicate Err3-positive γMNs and Err3-negative αMNs, respectively. The three Err3-negative αMNs are larger than the two Err3-positive γMNs. Scale bar in (**B**) refers to (**A**,**C**,**D**). (**E1**,**E2**) Enlarged images of the area enclosed by a square (yellow interrupted line) in (**D**) at two different Z levels. The Z level difference between (**E1**) and (**E2**) is 1 μm. Arrows indicate VGLUT1-positive terminals that are in close apposition to an Err3-negative αMN. The average diameter of the Err3-negative αMN ((**E1**); #) is 28 μm, and that of the Err3-positive γMN ((**E2**); *) is 17 μm. Scale bar in (**E2**) refers to (**E1**). (**F1**–**H1**,**F2**–**H2**) Successive images showing immunoreactivities for Err3 and ChAT. (**H1**,**H2**) show successive merged images. The Z levels of (**F1**–**H1**,**F2**–**H2**) are separated by 3 μm to show the nucleoli of Err3-positive γMN and Err3-negative αMNs, respectively, for the accurate measurement of cell size. The filled and two open arrowheads indicate anErr3-positive γMN and Err3-negative αMNs, respectively. The average diameters of two Err3-negative αMNs (upper and lower open arrowheads) are 18 and 17 μm, respectively, and that of an Err3-positive γMN (a filled arrowhead) is 20 μm. Scale bar in (**F2**) refers to (**F1**,**G1**,**G2**,**H1**,**H2**). Adapted from [12].

**Figure 3:**
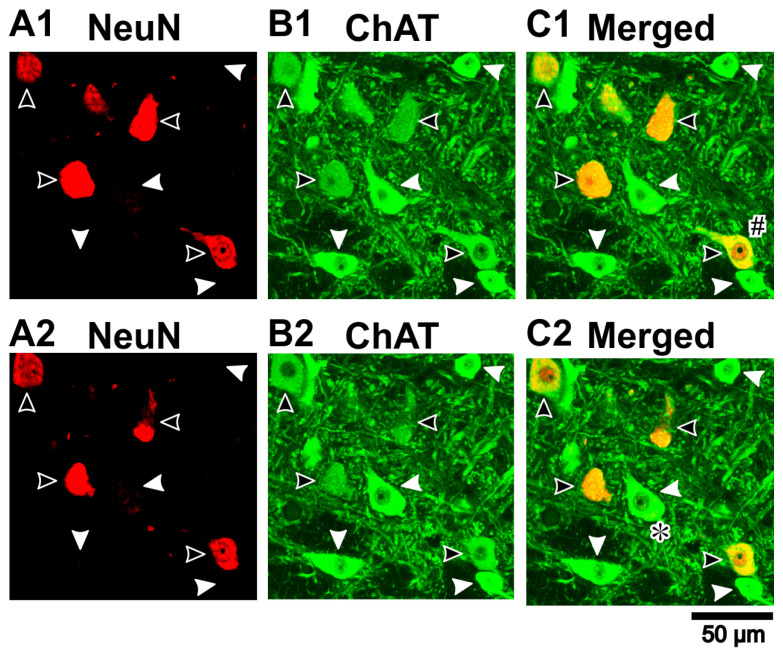
Immunohistochemical expression of ChAT and NeuN in the dorsolateral TMN MNs. (**A1**–**C1**,**A2**–**C2**) Successive confocal images showing immunoreactivities for NeuN (**A1**,**A2**) and ChAT (**B1**,**B2**). (**C1**,**C2**) show merged images. The Z levels of (**A1**–**C1**,**A2**–**C2**) are separated by 1 μm to show the nucleoli of the NeuN-positive αMN ((**C1**), #) and NeuN-negative γMN ((**C2**), *), respectively, for the accurate measurement of cell size. Open and filled arrowheads indicate NeuN-positive αMNs and NeuN-negative γMNs, respectively. NeuN-positive αMNs appear as small as NeuN-negative γMNs. Scale bar refers to all panels. Adapted from [12].

**Figure 4:**
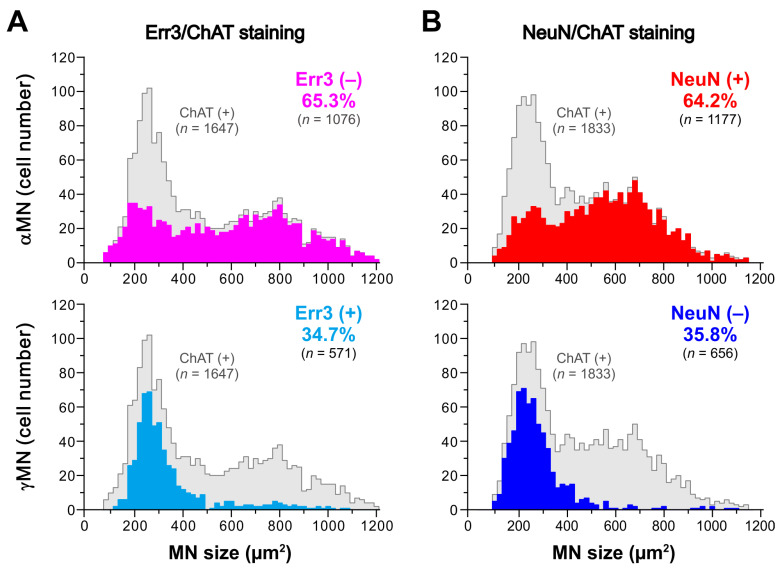
Size distributions of αMNs and γMNs in the dorsolateral TMN of rats. (**A**,**B**) Frequency distributions of cell sizes of αMNs and γMNs, which are identified by immunoreactivities for Err3 (**A**) or NeuN (**B**). The gray columns show all MN distributions, whereas the colored columns show the distributions of αMNs (upper panel) and γMNs (lower panel). αMNs display a bimodal size distribution. The size distribution of γMNs is unimodal and almost the same as that of the smaller αMNs. The size of MNs is represented as the cross-sectional area. Err3-negative αMNs display the bimodal size distribution with the two peaks at 180–200 and 800 μm^2^ (upper panel in (**A**)). Err3-positive γMNs display the unimodal size distribution with a peak at 260 μm^2^ (lower panel in (**A**)). NeuN-positive αMNs display the bimodal size distribution with the two peaks at 260 and 680 μm^2^ (upper panel in (**B**)). NeuN-negative γMNs display the unimodal size distribution with a peak at 220 μm^2^ (lower panel in (**B**)). Modified from [12].

Similar to those spinal MNs, ChAT-positive MNs in the TMN could be classified into αMNs and γMNs based on the immunoreactivities for NeuN and Err3 (Figure 2 and Figure 3). However, their size distribution was quite different from the bimodal distribution of the two populations of αMNs and γMNs in spinal motor nuclei. As shown in Figure 2(F1–H1), one Err3-positive γMN (filled arrowhead) and two Err3-negative αMNs (open arrowhead) were included in a section, and they were very closely located, allowing for a direct comparison in size. To accurately measure individual cell sizes, confocal images were taken at two different Z levels where the nucleoli of individual cells could be seen (Figure 2(F1–H1,F2–H2), respectively). A similar size distribution of NeuN-positive αMNs (open arrowhead) and NeuN-negative γMN (filled arrowhead) was observed, as shown in Figure 3. Thus, there were αMNs which were as small as γMNs in TMN, in contrast to the case with spinal MNs. Frequency distributions of cell sizes of αMNs and γMNs identified by using Err3/ChAT staining and NeuN/ChAT staining are presented in Figure 4. There were two populations of αMNs, a small-sized group of which was as small as γMNs in the TMN (Figure 4), in contrast to spinal αMNs.

More recently, Gfrα1 and Hb9 were identified as being expressed predominantly in αMNs and γMNs in the lumbar spinal cord, respectively [14]. Subsequently, the markers of MMP9, chondrolectin (Chodl) and Errβ [10] and those of wnt7a and 5-HT_1d_ [15,16] were found to be expressed in αMNs and γMNs, respectively, although MMP9, chondrolectin and Errβ were not always expressed in all αMNs. Additionally, osteopontin (Osteop) was also found as a marker for αMNs [17]. These molecular markers are summarized in Table 1.

## 3. Development of αMNs and γMNs

Membrane properties and firing patterns in spinal αMNs drastically change around at birth, and αMNs can display a train of spikes in response to current pulses at postnatal days 1–3 [21,22]. Subsequently, the repetitive firing frequencies increase along with the development during the first two postnatal weeks, resulting in firing patterns that are similar to mature patterns [23]. Such increases in repetitive firing frequency along with the development of αMNs are considered to be mediated by increases in the density of existing ion channels rather than by the appearance of new ion channels [21]. Indeed, the electrophysiological classification of αMNs in the dorsolateral TMN of postnatal day 7–12 rats [11], based on the presence of different subsets of ionic currents, was in good agreement with the classifications made in mature rats [24] or guinea pigs [25].

**Figure 5:**
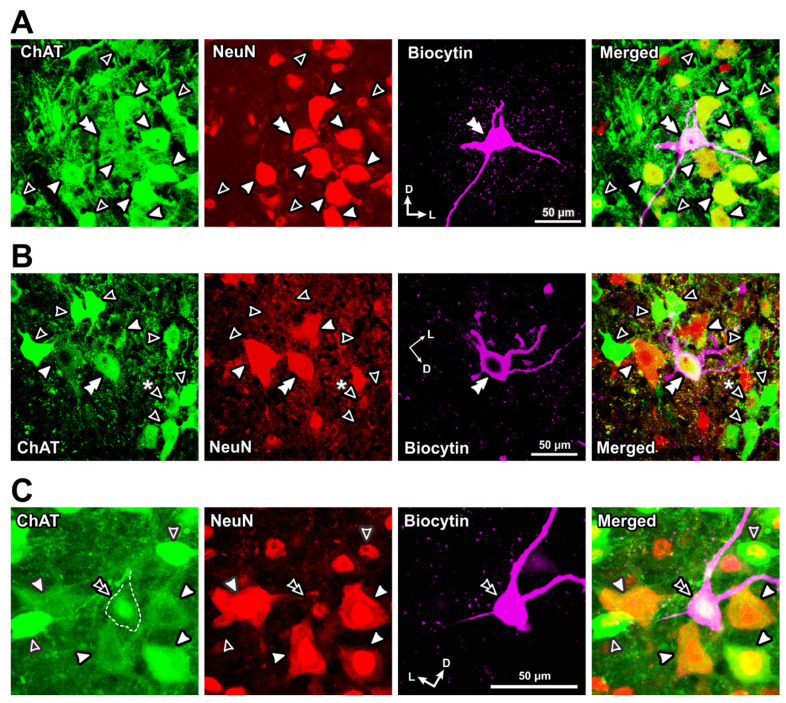
Immunohistochemical staining in Type I αMNs, Type II αMNs and γMNs. (**A**) Confocal images showing immunoreactivities for ChAT (green), NeuN (red) and biocytin (pink). Merged, a merged fluorescence image. Fluorescence signals for ChAT, NeuN and biocytin were obtained using Alexa Fluor 647, Cy3 and Alexa Fluor 488, respectively. A double filled arrowhead indicates the biocytin-labeled recorded neuron that was identified as ChAT-positive and NeuN (N+, C+) αMN. Filled arrowheads indicate ChAT-positive and NeuN (N+, C+) αMNs. Open arrowheads indicate ChAT-positive and NeuN (N+, C−) γMNs. (**B**) A double filled arrowhead indicates a biocytin-labeled recorded neuron that was identified as ChAT-positive and NeuN (N+, C+) αMN. Filled and open arrowheads indicate ChAT-positive with NeuN (N+, C+) αMNs and ChAT-positive with NeuN (N−, C−) γMNs, respectively. Open arrowhead with asterisk indicates a ChAT-positive and NeuN (N+, C−) γMNs. (**C**) A double open arrowhead indicates a biocytin-labeled recorded neuron that was identified as ChAT-positive and NeuN (N+, C−) γMN. Filled arrowheads indicate ChAT-positive and NeuN (N+, C+) αMNs. Open arrowheads indicate ChAT-positive and NeuN (N+, C−) γMNs. Modified from [11].

It is known that, regardless of αMNs or γMNs, all MNs express Err3 in their nuclei in the early postnatal stages, and the selective expression of Err3 in γMNs gradually occurs over the first two weeks after birth [9]. On the other hand, it is also known that during early postnatal periods to postnatal day 20, ~30% of γMNs weakly express NeuN, especially in their nuclei, while 100% of αMNs strongly express NeuN both in the nucleus and cytoplasm after postnatal day 0 [14]. Thus, in αMNs, the expression of NeuN has already been upregulated not only in the nucleus but also in the cytoplasm at postnatal day 0, while that of Err3 is downregulated along with the postnatal development over the first two weeks after birth. By contrast, in γMNs, NeuN is downregulated along with the postnatal development to postnatal day 20, whereas Err3 is maintained. Therefore, only after the first two weeks after birth, αMNs and γMNs in a spinal motor nucleus become molecularly distinguishable by the differential expression of NeuN and Err3 [9]. In a whole-cell patch-clamp recordings performed using rats at postnatal days 7–12 [11], consistent with these previous reports, almost all MNs in the trigeminal jaw-closing motor nucleus were Err3-positive, whereas MNs displayed three differential immunoreactivities for NeuN. Subsequently, it was possible to classify MNs based on the immunoreactivity to NeuN (Table 2). Consistent with the previous reports [9,12,14], the first type of MNs that displayed the prominent immunoreactivity for NeuN not only in their nucleus but also in their cytoplasm, namely NeuN (N+, C+) MNs, were classified as αMNs (Figure 5A–C; filled arrowhead), and the second type of MNs that displayed no immunoreactivity for NeuN in the nucleus and cytoplasm, namely NeuN (N−, C−) MNs, can be classified as γMNs (Figure 5B; open arrowhead). In contrast, the third type of MNs that showed the relatively weak immunoreactivity for NeuN only in the nucleus but not in the cytoplasm, namely NeuN (N+, C−) MNs, can also be classified as γMNs (Figure 5C; open arrowhead), as revealed in the previous report [14]. Thus, regardless of whether they were neonatal or adult, αMNs were invariably immunopositive for NeuN both in the nucleus and cytoplasm, whereas NeuN immunoreactivity in γMNs is downregulated in the cytoplasm first and subsequently in the nucleus along with postnatal development. As proposed previously [15,16], wnt7a or 5-HT_1d_ could be useful for further characterizing the electrophysiological properties of γMNs.

In rats, the transition of motor pattern from suckling to chewing occurs around postnatal day 12, and the acquisition of mature mastication occurs around postnatal weeks 2–3 [26,27]. As γMNs play an important functional role in the isometric contraction during chewing foods [4], the development of γMNs is likely to be closely related with the transition of motor pattern from suckling to chewing. Then, the downregulation of Err3 expression selectively in αMNs and that of NeuN expression selectively in γMNs of the dorsolateral TMN would be achieved around postnatal weeks 2–3. At such ages, however, patch-clamp recordings of MNs using brain slices are very difficult because of the much lower viability of MNs due to the severance of many dendrites, especially of αMNs, extending in every direction.

## 4. MNs in the Dorsolateral TMN

As reported previously, MNs in the dorsolateral TMN consist of 65% αMNs (Err3-negative/NeuN-positive MNs) and 35% γMNs (Err3-positive/NeuN-negative MNs) [12]. The size distribution of αMNs was bimodal, whereas that of γMNs was almost the same as that of small αMNs, indicating the presence of αMNs as small as γMNs in the dorsolateral TMN [12]. In a previous study [11], the electrophysiological and morphological characteristics of αMNs and γMNs in the dorsolateral TMN were investigated using whole-cell patch-clamp recordings in combination with immunohistochemical staining with anti-Err3 and anti-NeuN antibodies to identify whether the recorded neuron is αMN or γMN.

**Figure 6:**
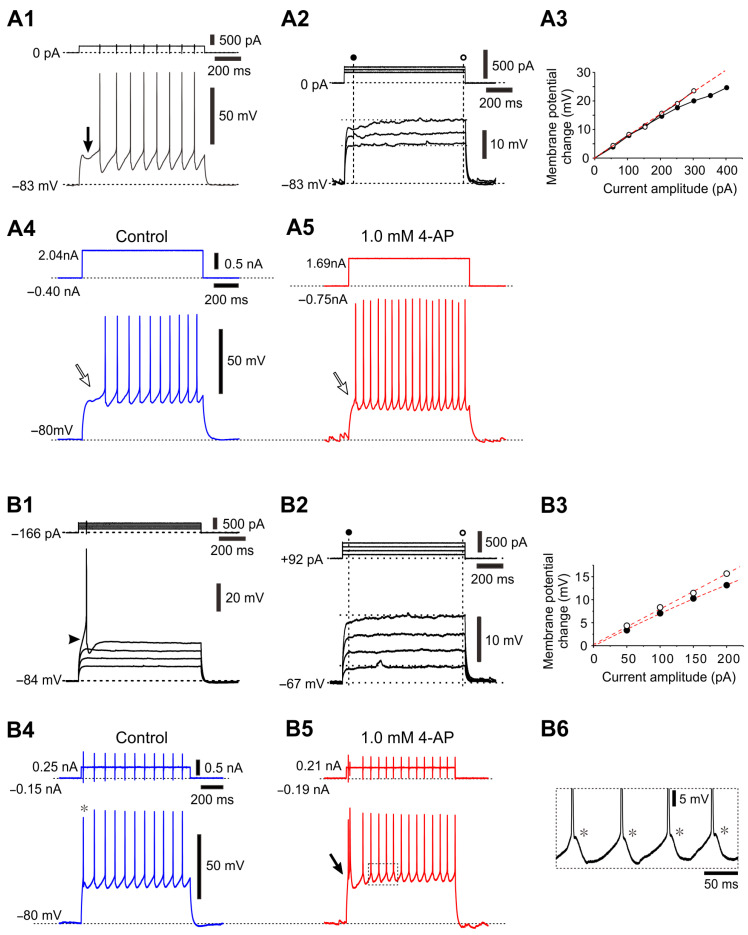
Electrophysiological properties of Type I and Type II αMNs. (**A1**) A spike train induced by injection of depolarizing current pulses in a type I αMN (see Figure 5A) at a resting membrane potential of –83 mV. A hyperpolarizing notch which causes a delay in the occurrence of the 1st spike (arrow). (**A2**) Subthreshold membrane potential responses in response to depolarizing current pulses applied at –83 mV. (**A3**) A relationship between the depolarizing current pulse amplitudes and the membrane potential changes measured 60 ms after the pulse onset ((**A2**), filled circles) and that measured 10 ms before the pulse offset ((**A2**), open circles), showing an early outward rectification. (**A4**,**A5**) Spike trains induced in a Type I αMN by injection of depolarizing current pulses at –80 mV before and during application of 4-AP (0.5 mM). The delay of the 1st spike ((**A4**), an open arrow) was almost abolished by 4-AP ((**A5**), an open arrow). (**B1**) A spike induced by injection of depolarizing current pulses in a Type II αMN (see Figure 5B) at –84 mV. An arrowhead indicates an LTS-like response (depolarized more than the level of the passive response as shown with a dotted line). (**B2**) Subthreshold membrane potential responses in response to depolarizing current pulses applied in the same Type II αMN at –67 mV. (**B3**) A relationship between the depolarizing current pulse amplitudes and the membrane potential changes measured 50 ms after the pulse onset ((**B2**), filled circles) and that measured 10 ms before the pulse offset ((**B2**), open circles), showing a less prominent early outward rectification compared to (**A3**). (**B4**,**B5**) Spike trains evoked by injection of depolarizing current pulses in a presumed type II αMN at –80 mV before and during application of 4-AP (1 mM). A filled arrow indicates bursts caused by LTS (**B5**). (**B6**) The enlargement of the portion of the trace enclosed by a rectangle in (**B5**) showing spike-ADPs or LTSs underlying spike generation (*). Modified from [11].

As summarized in Table 2, NeuN-positive αMNs in the dorsolateral TMN were classified into two subtypes, Type I and Type II αMNs: Type I αMNs had a relatively larger cell body and displayed a 4-AP-sensitive delayed spiking (Figure 6A recorded from Figure 5A), while Type II αMNs had a relatively smaller cell body and displayed a low-threshold Ca^2+^ spike (LTS) and a less prominent 4-AP-sensitive response (Figure 6B; arrowhead and arrow; recorded from Figure 5B). The presence of two types of αMNs in the dorsolateral TMN found in this study using juvenile rats at postnatal days 7–12 [11] was consistent with the findings made in the TMN of adult rats or guinea pigs, in which MNs displayed either Ni^2+^-sensitive LTS [24] or 4-AP-sensitive delayed spiking [25]. A similar classification of αMNs based on T-type Ca^2+^ currents and A-type K^+^ currents has been reported in rat abducens motoneurons in brainstem slice preparations obtained from postnatal days 1 to 13 Wistar rats [28].

**Figure 7:**
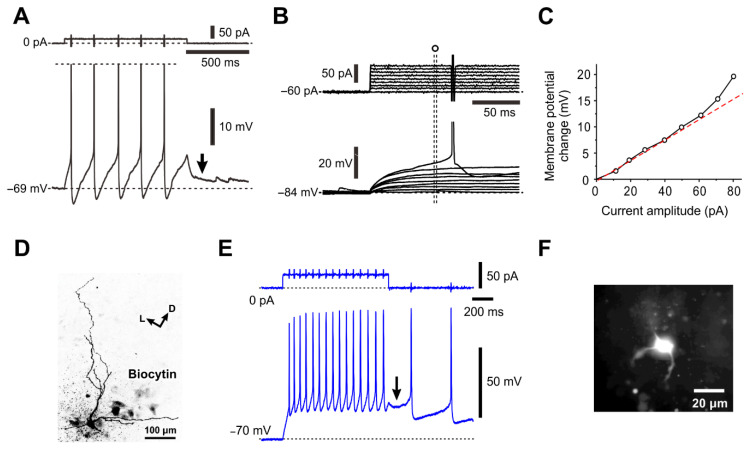
Electrophysiological properties of γMNs. (**A**) A spike train (spikes are truncated) induced in response to a depolarizing current pulse applied in a γMN (see Figure 5C) at –69 mV. A pulse-ADP is observed after the pulse offset (arrow). (**B**) Subthreshold membrane potential responses to depolarizing current pulses applied in the γMN at –84 mV. (**C**) A relationship between the depolarizing current pulse amplitudes and the membrane potential changes measured at 67–70 ms after the pulse onset (open circles), showing a superlinear I-V relationship of the subthreshold membrane responses in contrast to that seen in αMN. (**D**) The recorded neuron (**A**–**C**) labeled with biocytin showing sparse arborizations of primary dendrites (see Figure 5C). (**E**) An injection of a depolarizing current pulse at –70 mV to a neuron induced a spike train, followed by a pulse-ADP (arrow) that caused further spikes. (**F**) A lucifer yellow image of the recorded neuron (**E**) that was electrophysiologically identified as γMN, showing sparse arborizations of primary dendrites. Modified from [11].

**Figure 8:**
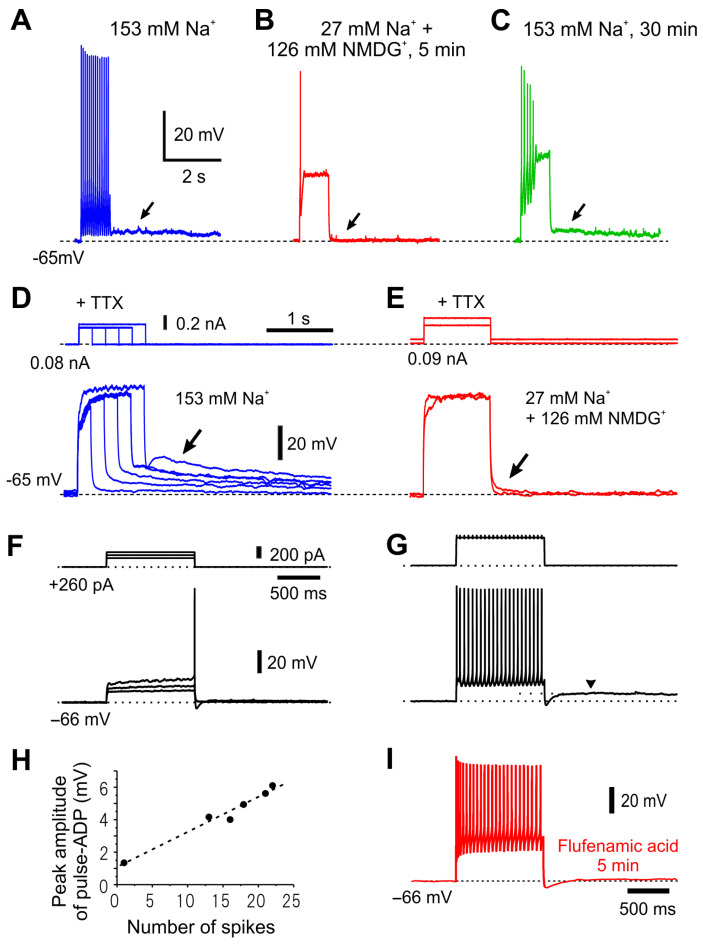
The pulse-ADP is mediated by Ca^2+^-dependent cation channels. (**A**–**E**) Long-lasting pulse ADP induced in a presumed γMN. (**A**) In response to a current pulse injection in artificial cerebrospinal fluid (aCSF) containing 153 mM Na^+^, a pulse-ADP that lasted for more than 5 s (arrow) was caused. (**B**) Abolishment of the pulse-ADP (arrow) by substitution of 126 mM Na^+^ with the equimolar N-Methyl-D-glucamine (NMDG)^+^. (**C**) Restoration of the pulse-ADP (arrow) following washout of NMDG^+^ with the original aCSF. (**D**) In the presence of TTX (1 μM), amplitudes of pulse-ADPs increased (arrow) with increases in the duration or the amplitude of the depolarizing current pulse. (**E**) In the presence of TTX, the pulse-ADP was abolished (arrow) by substitution of 126 mM Na^+^ with the equimolar NMDG^+^. (**F**,**G**) Voltage responses to depolarizing current pulses applied in a presumed γMN at –66 mV. Note that the subthreshold membrane responses displayed a superlinear I-V relationship. (**F**) As the number of spikes was increased by increasing the current pulse intensities, the amplitudes of pulse-ADPs increased. Arrowheads indicate pulse-ADPs (**G**). (**H**) A linear relationship between the spike numbers and the peak amplitudes of pulse-ADP. (**I**) An application of 10 μM flufenamic acid abolished the pulse-ADP which was induced following a train of spikes evoked by a constant depolarizing current pulse applied in the same γMN at –66 mV. Modified from [11].

In a previous study [11], it was demonstrated that, in the MNs which were either NeuN (N−, C−) or NeuN (N+, C−) (Figure 5B,C), both equally had smaller cell bodies and displayed a characteristic pulse-afterdepolarization (pulse-ADP) (Figure 7A,E; downward arrow). These MNs seems to be mostly γMNs, in consideration of the postnatal downregulation of NeuN in γMNs [14]. In addition, there were non-cholinergic mall-sized neurons, presumably GABAergic interneurons, which displayed a prominent LTS but none of I_KA_, pulse-ADPs and persistent inward current (Table 2). The profile of the size distribution of Type I and II αMNs and γMNs revealed by this study was in good agreement with the previous findings [12]. Similar differences in the firing pattern were also observed between a larger and smaller TMN in a previous study showing rank-ordered recruitment of trigeminal MNs (see Figure 6; [8]). These results suggest that the intrinsic excitability increases in an ascending order: type I αMNs < type II αMNs < γMNs.

## 5. Ionic Mechanism Underlying Pulse-ADP and Its Functional Implications

The ionic mechanism underlying pulse-ADP, which characterizes the firing pattern of γMN, has been investigated in detail. Substitution of extracellular Na^+^ with NMDG^+^ almost completely abolished the pulse-ADP evoked at the offset of the current pulses regardless of whether in the presence (Figure 8A–C) or absence of tetrodotoxin (TTX) (Figure 8D,E), suggesting the involvement of a cationic current largely carried by Na^+^ influx in the generation of the pulse-ADP. Furthermore, the amplitude of the pulse-ADP increased as the duration (or the amplitude) of the current pulses (Figure 8D) or the number of spikes evoked during the current pulse (Figure 8H) were increased, suggesting the Ca^2+^-dependent nature of the cationic current. Finally, an application of flufenamic acid, Ca^2+^-dependent cation channel blocker, abolished the pulse-ADP (compare Figure 8G,I), clearly indicating that the pulse-ADP was mediated by Ca^2+^-dependent cationic current as the ADP-induced bursting has been demonstrated to be blocked by flufenamic acid in respiratory MNs [29] or in cortical pyramidal cells [30]. Because the peak potential level of the fully activated pulse-ADP was around −45 mV (Figure 8D), the reversal potential for the cationic current is near −45 mV, given the involvement of the cationic current in the generation of the pulse-ADP. If this is the case, the cationic channel is weakly selective for K^+^ over Na^+^. Then, such a cationic current can facilitate spike repolarization to −45 mV by acting as an outward current, whereas it can generate ADP at the potentials that are more negative than –45 mV by acting as an inward current. There are at least three distinct cationic currents mediated by ionic channels with a similar ionic selectivity, which are either activated by Ca^2+^ dependently [31] or independently [32] or through the G-protein-coupled receptor [33,34]. Therefore, if the pulse-ADP in γMNs is enhanced by activation of some metabotropic receptors, the enhanced long-lasting pulse-ADP might cause a “tonic-like persistent firing”. This tonic drive of Ia synaptic inputs by γMNs may be important especially for αMNs in the TMN to induce temporal summation instead of spatial summation of Ia-EPSPs, because the number of synapses between single group Ia afferent and single αMN in the TMN is much smaller [35,36] than in those between single group Ia afferent and single αMN in the spinal cord [37,38]. Indeed, the spatial summation of Ia-EPSPs would easily activate αMNs in the spinal cord, while the temporal summation of Ia-EPSPs would be required to activate jaw-closing αMNs in the TMN. This can be supported by the difficulty and easiness in evoking H-reflex in the resting and the slight clenching condition of jaw-closing muscles, respectively, as reported previously [39]. An aberrant activation of such cationic current would cause hyper-excitation of γMNs, which can lead to the generation of γ-rigidity [40], probably responsible for bruxism or oral dyskinesia in patients with Parkinson disease.

## 6. Functional Implication of Morphological and Electrophysiological Differences between Type I and II αMNs in the Orderly Recruitment of MNs

In the spinal cord, αMNs were classified into two subtypes based on the firing patterns: delayed firing and immediate firing, representing fast and slow αMNs, respectively [10]. However, there are three types of motor units: FF, FR and S. Therefore, delayed-firing fast αMNs may be responsible for FF and FR motor units. NeuN immunopositive delayed-firing fast αMNs can be further separated into two groups by the immunoreactivities for chondrolectin and MMP-9. Chondrolectin-negative delayed-firing αMNs had a relatively lower rheobase and a larger AHP relaxation time constant compared to chondrolectin-positive ones, while MMP-9 immunonegative delayed-firing αMNs had a relatively lower rheobase and a smaller input conductance compared to MMP-9 immunopositive ones. Based on these observations, it was postulated that delayed-firing fast αMNs responsible for FF and FR motor units may be those immunopositive to chondrolectin/MMP-9 and those immunonegative to chondrolectin/MMP-9, respectively [10].

On the other hand, in the TMN, αMNs displayed a clear bimodal distribution of their sizes, and showed two distinct firing patterns [12]. Motor units in a jaw-closing motor system may be distinct from the locomotor or limb motor systems, as is reflected in the differences in the muscle fiber components between limb muscles and jaw-closing muscles [41,42,43]. Limb muscles in rats consist of a mixture of type I fibers with slow-twitch myosin heavy chain isoform and type II fibers with fast-twitch myosin heavy chain isoform [42]. In contrast, masseter muscles in rats consist of only type II fibers and consequently exhibit high ATPase activity [41,43]. However, histoenzymatic stainings for succinic dehydrogenase, myoglobin and ATPase activities revealed that masseter muscles are composed of no pure white fibers but red and intermediate fibers [44,45,46]. Thus, in a jaw-closing motor system, there are two types of motor units—FR and S—and Type I and Type II αMNs are responsible for FR and S, respectively.

Consistent with the higher input resistance of Type II αMNs compared with Type I αMNs, the mean size of the somata was significantly smaller in Type II αMNs than in Type I αMNs [11]. Furthermore, the spike threshold in Type II αMNs was significantly lower compared to that in Type I αMNs, not only due to the higher input resistance but also due to the presence of LTS which is presumably mediated by Ni^2+^-sensitive transient T-type Ca^2+^ current as previously demonstrated in abducens MNs [28]. Thus, the input resistance, the soma size and the spike threshold were in favor of the orderly recruitment from Type II αMNs to Type I αMNs. Furthermore, the immediate or fast phasic firing followed by tonic firing observed in Type II αMNs was in contrast or complementary to the delayed tonic firing in Type I αMNs. Thus, these differences in the intrinsic membrane properties and the subsequent firing pattern between the two types of αMNs in the TMN may be the bases for the rank-ordered recruitment of αMNs in the TMN, although the group Ia synaptic input is also known to be in favor of the rank-ordered recruitment of MNs [47]. Although the spike afterhyperpolarization was not measured in the previous study, the spike duration was significantly longer in Type II than in Type I αMNs [11], consistent with the classical classification of slow and fast MNs [48,49]. In view of the differences in the excitability between Type I and Type II αMNs, Type II αMNs are likely to innervate the slow Type I or Type IIA muscle fibers, while Type I αMNs are likely to innervate the Type IIB muscle fibers of jaw-closing muscles.

## 7. Conclusions

In this review, we summarized the recent advances in research on the classification of αMNs and γMNs in the spinal cord and TMN based on the various molecular markers and electrophysiological membrane properties. Functional roles and significance of subtypes of αMNs with distinct firing properties were discussed from the aspects of the rank-order recruitment and the motor unit. Distinct firing properties and detailed membrane properties of γMNs were reported first in TMN.

## Figures and Tables

**Table 1:** Summary of molecular markers of motoneuron subtypes.

		NeuN [11,12]	Err3 [9,12]	Hb9 [14]	Gfrα1 [14]	Wnt7a [15]	5HT_1d_ [16]	Osteop. [17]	VGlut1 [12]	Err2 [18]	Chodl [10,18,19]	MMP9 [10,20]
αMN	Larger group	+	–	+	–	–	–	+	+	–	±	±
	Smaller group	+	–	±	±	–	–	+	+	+	–	–
γMN		–	+	–	+	+	+	–	–	–	–	–

**Table 2:** Electrophysiological classification of neurons and their sizes in the dorsolateral TMN. Modified from [11].

	αMN				γMN		non-MN
	Type I		Type II				
	Total	ChAT(+) NeuN (C+,N+)	Total	ChAT(+) NeuN (C+,N+)	Total	ChAT(+) NeuN (C−,N±)	ChAT(−) NeuN(+)
Number of cells	14	6	22	12	18	8	11
Size (µm) Long axis	30 ± 7	31 ± 5	24 ± 7	26 ± 6	19 ± 4	22 ± 2	12 ± 4
Short axis	23 ± 4	25 ± 3	17 ± 4	17 ± 5	15 ± 3	16 ± 3	12 ± 3
4-AP-sensitive K^+^ current (transient I_KA_)	++		+		−		−
Delayed firing	+		−		−		−
Low-threshold Ca^2+^ spike (LTS)	−		+		−		++
Apamin-sensitive K^+^ current	−		+		−		−
Pulse afterdepolarization (pulse-ADP)	−		−		+		−
Persistent inward current	−		−		+		−

## Data Availability

Data are contained within the article.
